# Needs to connect to urban nature in female university students from Southern Germany: a mixed methods concept mapping study

**DOI:** 10.3389/fpubh.2026.1758383

**Published:** 2026-03-17

**Authors:** Dorothea M. I. Schönbach, Ximena Tiscareno-Osorno, Tadhg E. MacIntyre, Stephen Smith, Deirdre MacIntyre, Yolanda Demetriou

**Affiliations:** 1 Department of Rehabilitation and Sports Medicine, Hannover Medical School, Hanover, Germany; 2 TUM School of Medicine and Health, Technical University of Munich, Munich, Germany; 3 Psychology Program, Faculty of Business, University of Europe for Applied Sciences, Berlin, Germany; 4 Innovation Value Institute, Maynooth University, Maynooth, Co. Kildare, Ireland; 5 Research Ireland Insight Centre for Data Analytics, Maynooth University, Maynooth, Co. Kildare, Ireland; 6 Institute of Child Education and Psychology, Europe, Maynooth, Co. Kildare, Ireland; 7 Institute of Sports Science, University of Tübingen, Tübingen, Germany

**Keywords:** adults, mixed methods, needs assessment, urban nature connectedness, women

## Abstract

Mental health problems are a major global health concern today. Psychological well-being, such as mental health, can be improved through nature connectedness. Nature connectedness is a co-benefit of planetary health. However, access to nature, along with sufficient exposure to it, which promotes nature connectedness, is a challenge for urban inhabitants. Therefore, this study aims to identify the needs required to connect with urban nature. Between June and August 2021, 152 female university students from Southern Germany participated in a mixed methods concept mapping study. Urban nature connectedness was rated as relatively low. Overall, nine different needs related to urban nature connectedness were identified. In line with previous research, we categorized these needs into three domains (situational contexts: *n* = 7, individual differences and psychological states: *n* = 1), respectively. The predominance of the situational contexts domain suggests that urban nature connectedness is a state rather than a trait. Only one need (i.e., accessibility) was rated as important, thereby suggesting that a mere summary of needs—without considering their respective importance—is insufficient to draw solid conclusions for practical implications. This study provides unique insights into the development of urban nature connectedness among female individuals. Our findings should be considered in future research on the operationalization of the construct, the development and validation of a robust measuring instrument, and the design of an intervention. In addition, our findings can inform policymakers and city planners about the optimization of urban design to improve urban nature connectedness and, consequently, health among urban inhabitants.

## 1 Introduction

Currently, 55% of the world’s population lives in urban areas, and this percentage is expected to rise to 68% by 2050 (1). In Europe, more than 60% of urban inhabitants live in areas without sufficient green spaces (2). The World Health Organization recommended in 2017 that every urban inhabitant should have access to a green space of at least 0.5–1 hectare within 300 meters of their home (i.e., roughly a 5-min walk as “the crow flies”) (3). A more recent recommendation from 2021 suggested that at least three trees should be visible from one’s home, that trees or other vegetation should cover at least 30% of the neighborhood, and that a park or green space should be accessible within 300 meters (4). By 2030, the United Nations calls for universal access to sufficient, inclusive, and safe urban green spaces—particularly for female individuals—under Target 11.7 of the established Sustainable Development Goals (5).

Concerning psychological health, mental health problems are globally prevalent in approximately 11% of the world’s population (i.e., 792 million people) (6). This underlines the urgent need to improve mental health globally. In Target 3.4 of the established Sustainable Development Goals, the United Nations calls for an improvement in mental health and well-being by 2030 (5). Mental health (e.g., symptoms of depression and stress levels) improves in proportion to the availability of urban green space (7, 8). Happiness (9), mood (10), and stress (11) are also positively influenced by exposure to urban nature. In addition, physical activity (12, 13), social cohesion (12, 13), depression (12, 13), self-reported health (12), and high blood pressure (13) are positively impacted by the urban nature dose (i.e., frequency and/or duration of exposure). However, the nature dose and the level of urbanization are negatively related (12).

The biophilia hypothesis describes an innate, genetic affinity of humans for nature from various perspectives, including esthetic, symbolic, cultural, biological, and psychological (14). It suggests that psychological health is related to nature through humans’ need to connect with it (15). For example, nature connectedness has been associated with enhanced mental health (i.e., decreased mental distress and lower odds of needing medical treatment for depression) (16), hedonic well-being (i.e., increased positive affect, life satisfaction, and vitality) (17), and eudaimonic well-being (i.e., personal growth) (18). Therefore, the biophilia hypothesis is one of the key theoretical frameworks behind the development of the construct of nature connectedness. As the process of operationalizing a definition of the multi-dimensional and complex construct of nature connectedness maintains (19–21), we define the dimensions of this construct as follows based on previous research (22–24), we define the dimensions of this construct as follows: (a) affective (i.e., emotions, such as enjoyment of nature), (b) experiential (i.e., direct interaction or experience, such as outdoor activities in blue or green spaces), (c) cognitive (i.e., knowledge, beliefs, attitudes, identity, values, and awareness, such as inclusion of nature in the self), (d) behavioral attitude (i.e., commitment, such as orientation toward nature), and (e) spiritual (i.e., philosophical, such as the meaning and purpose of being). In our recent systematic review, we provided the following preliminary definition of nature connectedness (21, p. 554): “People have a basic need of belonging, which can be satisfied by being subjectively and positively connected to nature. Being connected to nature includes being close to or one with the natural world.” [sic] In urban areas, nature connectedness is called urban nature connectedness – a construct whose conceptual clarity is still emerging. However, nature connectedness is reduced in urban areas (25–27). Regarding this loss of interaction with nature, a previous review introduced the concept of the extinction of experience, which considers the definition of nature (i.e., narrow vs. broad), the timing of loss (i.e., childhood vs. lifelong), and the focus (i.e., interaction vs. experience) (28). This loss has been shown to negatively affect health, well-being, emotions, pro-environmental attitudes and behavior, and satisfaction with nature (29). Consequently, (urban) nature connectedness is a co-benefit of planetary health. A need for action is required, as the rising percentage of urban inhabitants appears to have restricted access to nature and the nature dose, which, in turn, may lead to a reduced level of urban nature connectedness, increased health risks, and a higher likelihood of environmentally unfriendly behavior.

To address potential inequalities (Goal 10 of the United Nations’ Sustainable Development Goals) (5), when compared to rural inhabitants, it is essential to understand what factors influence urban nature connectedness and what is needed to reconnect urban inhabitants with nature (e.g., through the development of an intervention). A review of the pathways to nature connectedness identified three domains: (a) situational contexts, (b) individual differences, and (c) psychological states (30). However, little is known about factors predicting urban nature connectedness to date. In a sample from Australia, for example, previous nature experiences and the duration of current nature exposure were positively correlated (31). Our previous study conducted in Germany found that living in a rural area was a negative predictor of urban nature connectedness, whereas spending more hours per week in nature and engaging in outdoor activities were positive predictors (32). Another study from Israel found that having previously lived in a rural area (e.g., during childhood) was positively related to two dimensions of nature connectedness (i.e., cognition and affection) (27). In addition, a study from Japan revealed that, independent of the level of urbanization, nature connectedness was positively associated with perceived values of nature (26). This inconsistency in the published literature—reflected in conflicting findings—and the limited number of studies might be explained by the fact that the construct of urban nature connectedness has not been clearly operationalized yet. Consequently, the lack of established construct-related clarity makes it difficult to develop instruments to measure this construct across different age groups (i.e., children, adolescents, and adults). In our recent systematic review (21), we found only one validation study of an explicit instrument designed to measure urban nature connectedness. This instrument was utilized with preschool children from China and assessed four dimensions: (a) awareness of nature, (b) responsibility toward nature, (c) empathy for nature, and (d) enjoyment of nature (33).

To the best of our knowledge, we are not aware of any research following a participatory approach to gain comprehensive insights into the components needed to develop and promote urban nature connectedness, which justifies conducting research on this topic. Therefore, this concept mapping study aimed to identify what university students need to connect with urban nature (i.e., a needs assessment). The factors we identified as necessary for fostering urban nature connectedness can contribute to a clearer operationalization of this construct, which, in turn, can be used to develop and validate a robust measurement instrument, design an intervention to improve urban nature connectedness, and evaluate its effectiveness. Therefore, this study has the potential to provide considerable added value for the health of urban inhabitants. Overall, our endeavor is in line with Goals 3, 10, 11, and 13 of the United Nations’ Sustainable Development Goals (5).

## 2 Materials and methods

**Figure 1:**
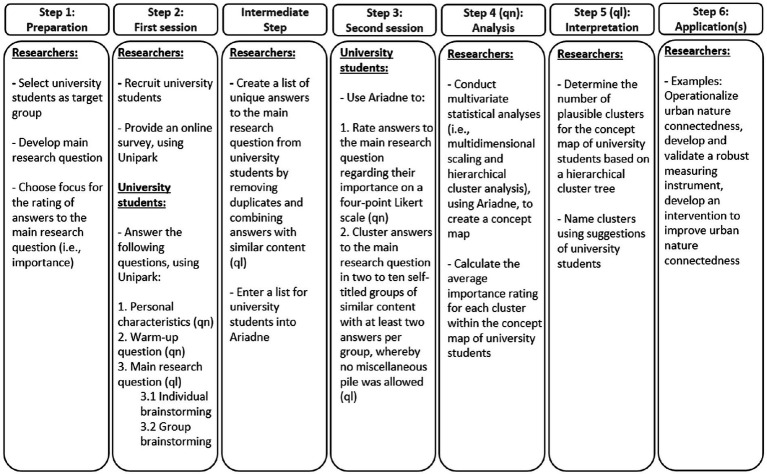
Visualization of our concept mapping approach. Ariadne = concept mapping software; ql = qualitative; qn = quantitative; Unipark = survey software.

For our needs assessment, we conducted a concept mapping study in which participants were the experts on their own needs. Concept mapping is an innovative mixed method approach (34) including a combination of quantitative and qualitative methodologies (35), as recommended in previous research (36). A pooled analysis of 69 concept mapping studies confirmed its high validity and reliability (37). It is suitable for complex and ambiguous subjects, issues characterized by various perspectives, and for establishing a common foundation or identifying priorities for future actions (38). The method is efficient, no special skills or knowledge are required to participate, and perspectives of all participants are treated equally (38). By using group processes (34), it adopts a participatory approach (34, 35). It is structured in six steps (39): (1) the researcher selects the target group, develops the main research question, and determines the focus for rating answers to the main research question (e.g., importance and/or feasibility), (2) the target group answers the main research question in their own language during individual and group brainstorming phases, (3) the target group rates the answers and organizes them into clusters of similar content, (4) the researcher creates concept map(s), (5) the researcher interprets the concept map(s), and (6) the researcher uses the concept map(s) for planning or evaluation purposes (e.g., needs assessment or intervention development). Figure 1 presents the concept mapping approach applied in this study, from its preparation to application(s) (i.e., steps 1–6).

### 2.1 Participants

At the Technical University of Munich, 190 students enrolled in the sixth semester of the Bachelor’s program in “Health Science,” who were attending the mandatory module “Health promotion programs,” were initially invited via e-mail to volunteer for this study between 10th June and 7th August 2021. Originally, we wanted to conduct our research among university students of both sexes. University students represent a critical population to study, as they experienced mental health issues and impaired well-being during the COVID-19 pandemic in Germany (40) and are generally capable of assessing their own nature connectedness (41). However, as only 14 male university students participated in our study, they had to be excluded from the analysis due to the small sample size and imbalanced distribution between the sexes. Therefore, the following description of the methods refers only to data collected from female university students. Female university students are also a critical population to study, as they were more frequently affected by major depressive or anxiety disorders compared to their male counterparts during the COVID-19 pandemic in Germany (40). In addition, previous research has suggested that female individuals might be more connected to nature compared to their male counterparts (30).

### 2.2 Data collection

Attendance in the course “Health promotion programs” was not mandatory. Therefore, university students were offered additional course credit in exchange for full participation in the study. Before participating in this online study, university students read and signed an online informed consent form. Only one university student refused to participate in the study. For this cross-sectional concept mapping study, a statement from an ethics committee was not required. According to the German Research Foundation, such approval is not needed when participants are fully informed about the study and when a study does not involve deception, a high level of emotions, stress, or traumatic experiences, exceptional risks (e.g., social or physical), or vulnerable groups (42). In accordance with institutional regulations, a statement from the ethics committee of the researcher’s institution is also not required for non-medical studies (43). At the time the study was conducted, the ethics committee of the researcher’s institution did not have a non-medical specialist group (43). Only 2 years after this study was conducted, a non-medical specialist group was established (i.e., in 2023) (43). The data collected were stored at the researcher’s institution where the study was conducted, meeting the high standards of data storage in Germany.

#### 2.2.1 First concept mapping session

In June 2021, the first concept mapping session was conducted during an online 90-min class. The students were randomly divided into two groups due to the large cohort size. Each group completed the survey using Unipark over two consecutive weeks during the semester. Each group was further randomly divided into smaller subgroups per week using breakout sessions. This grouping had no other purpose than to simplify the process. An experienced female researcher (DMIS) and four previously trained female assistants (LL, SM, AE, and PW) used Zoom to moderate the participating female university students. This procedure—where a female researcher or assistant supervised a group of female participants—was considered most appropriate to minimize the risk of reflexive bias by avoiding the mixing of female and male perspectives. A previous study analyzing interviewer effects found that respondents sometimes tend to provide answers similar to those of the interviewer, indicating social desirability (44). Another previous study concluded that female respondents benefit from gender-based interviewer–respondent matching, in line with the theory of liking, suggesting that data quality may improve when interviewers and respondents share characteristics (e.g., gender) (45). In our survey, the following items were included: (a) age, (b) sex (based on biological/physiological attributes), (c) zip code of residential area, (d) marital status, (e) parental status, (f) extent of media use, (g) religious affiliation, (h) dog ownership, (i) access to nature, (j) time (in days and h per week) spent in nature, (k) engagement in outdoor activities (e.g., walking, jogging, cycling, mountain biking, hiking, climbing, bouldering, horse riding, surfing, or diving), and (l) self-reported urban nature connectedness based on participants’ own understanding of the construct, rated on a four-point Likert scale with 1 = not at all, 2 = rather not, 3 = rather yes, and 4 = very much. Question (l) was a warm-up question and served as an icebreaker to introduce participants to the research topic “urban nature connectedness”: How connected are you to urban nature? Questions (a)-(l) produced quantitative data. The last question included in the survey was our main research question (m), which produced qualitative data: What do you need to connect to urban nature? To help participants who found it difficult to answer the main research question based on their own understanding of the construct, the following sub-questions were provided: (m_1_) Why and when do (not) you connect to urban nature? (m_2_) and What connects you to urban nature or what prevents you from connecting to urban nature? During the individual brainstorming phase, participants were encouraged to freely write down as many answers to the question (m) as they could think of, including factors that were already present or still missing. This was followed by a group brainstorming phase, in which each participant shared one unique answer to the question (m) at a time until all unique answers from every participant had been shared. Meanwhile, the listening participants reviewed the shared answers for comprehensibility to ensure clarity for all participants; if an answer was unclear, the participant who provided it was asked to offer further explanation. If participants were spontaneously inspired by another participant’s answer, they added new unique answers of their own, continuing this process until all needs for urban nature connectedness had been mentioned.

As a post-processing step following the first concept mapping session, DMIS created a list of all unique answers obtained during the brainstorming phase across all online sessions over the two consecutive weeks. As part of this process, DMIS used a qualitative approach. In doing so, DMIS removed duplicates and combined answers with similar content, which is in line with practical recommendations for this methodological approach to minimize the burden on participants (35, 39). SM reviewed the list. Any discrepancies that emerged during the review process were resolved through discussion between DMIS and SM. Following this, DMIS entered the list into Ariadne, a computer program—developed by Peter Severens (Talcott B. V.)—specifically designed for concept mapping studies. More details on how Ariadne was used are provided in this article whenever the program is mentioned. Finally, DMIS created a personal access link for each participant in an Excel file (RRID: SCR_016137) and prepared a detailed instruction manual for the second concept mapping session.

#### 2.2.2 Second concept mapping session

In July 2021, the second concept mapping session was conducted during an online 90-min class. DMIS used Zoom to supervise participants and to share the previously prepared detailed instruction manual. Each participant searched for and opened their personal link in the prepared Excel file to get access to Ariadne. First, participants were asked to rate each answer to the main research question, “What do you need to connect to urban nature,” from the list regarding its importance, using a four-point Likert scale: 1 = very unimportant, 2 = unimportant, 3 = important, and 4 = very important. This produced quantitative data. Second, participants were asked to inductively cluster the answers from the list into groups of similar content, in accordance with the following rules: (a) create a minimum of two and a maximum of 10 groups, (b) include at least two answers per group, (c) set a name for each group, and (d) avoid using “miscellaneous” as a group name. Consequently, participants followed a qualitative approach. Participants made their decisions on how to rate and cluster answers independently, without any influence from the supervising researcher. Unfortunately, we had technical issues when using Ariadne as too many participants entered their data at the same time. To reduce the risk of missing data, participants were asked to check the first and second tasks for completeness (i.e., to ensure that all answers had been rated and clustered). As technical issues persisted, the course cohort was randomly divided into two groups. Each group was asked to complete the two tasks unsupervised on their own time at home, outside of Zoom, within an assigned timeframe by August 2021.

### 2.3 Analysis

Quantitative personal and warm-up questions (i.e., items a-l) from the first concept mapping session were analyzed using IBM SPSS Statistics V.27 (RRID: SCR_016479) by merging data from 152 eligible female university students. For question (c), the zip code of the residential area was used to generate the following categories of urbanization levels: Rural: 2000–5,000 inhabitants, small town: 5000–20,000 inhabitants, medium-sized town: 20,000–100,000 inhabitants, and city: >100,000 inhabitants.

Qualitative answers to the main research question (m), “What do you need to connect to urban nature?”, obtained in the first concept mapping session and further processed in the second session, were also analyzed quantitatively. Only complete datasets for the rating and clustering tasks, respectively, were included in the analysis. This means that female university students were eligible for analysis only if they had rated and/or clustered all 98 answers to the main research question. Consequently, a sample size can be provided for female university students who were eligible for analysis in the second concept mapping session overall and a sample size for each of the two tasks, which were completed in this particular session. Overall, 142 female university students were eligible for analysis, thereof 135 for the rating task and 139 for the clustering task. The aforementioned technical issues resulted in missing data overall (*n* = 10), in the rating task (*n* = 17), and the clustering task (*n* = 13). To construct a concept map, multivariate statistical analyses (34)—including multidimensional scaling and hierarchical cluster analysis (34, 39)—were conducted using Ariadne. Based on the answers clustered by participants, a hierarchical cluster tree split the answers into 2 to 18 adequate clusters by default. A hierarchical cluster tree shows how clusters divide into two as the number of clusters increases or how clusters merge into one as the number of clusters decreases (38). DMIS examined all possible clusters presented in the hierarchical cluster tree. Based on the answers included in each cluster, DMIS determined the number of plausible clusters (i.e., answers representing similar content) for the concept map. This decision relied on subjective judgment (i.e., face validity). The output produced by Ariadne was a two-dimensional concept map, in which answers were arranged as dots (see Supplementary Figure S1). The imaginary distance between dots was determined by how often participants had clustered answers with similar content in the same group based on their own judgment—dots were closer when answers had been clustered more frequently in the same group and farther apart when clustered less often. The concept map represents a collective product based on the individual clusters of participants that included their provided answers. To ensure consistency of answers in the clusters of the concept map, DMIS, if necessary, inductively reassigned answers to an existing cluster (indicated by an arrow) or created a new cluster (indicated by a circle). SM and AE reviewed the decisions made by DMIS. Any discrepancies during the review process were resolved through discussion. Finally, DMIS named the clusters following the style of suggestions provided by female university students. This means that data interpretation followed a qualitative approach to ensure the plausibility of clusters, as relying solely on statistical significance does not necessarily guarantee that clusters contain answers representing similar content. Lastly, DMIS deductively categorized the clusters according to the dimensions of nature connectedness outlined in the introduction (i.e., affective, experiential, cognitive, behavioral attitude, and spiritual). XT-O reviewed DMIS’s categorization, and any discrepancies during the review process were resolved through discussion. The average importance rating for each cluster in the concept map was calculated based on the average rating of each included answer, with a value of ≥3.0 considered important. A standard deviation was also calculated for each cluster.

## 3 Results

### 3.1 First concept mapping session

Overall, the 152 participating female university students were aged 22.7 ± 2.8 years (see Supplementary Table S1). Most lived in a city (56.0%), followed by a medium-sized town (17.3%), a small town (15.3%), and a rural area (11.3%). The majority had access to nature (98.7%). They spent 4.5 ± 1.8 days and 14.4 ± 10.6 h per week in nature. A sizeable proportion of the cohort engaged in outdoor activities (87.5%). Participants reported relatively low urban nature connectedness (2.8 ± 0.7).

### 3.2 Second concept mapping session

In this section, quotes from the answers of female university students are provided in italics as examples.

#### 3.2.1 Concept map of female university students

The concept map of 139 female university students revealed nine clusters, representing 98 answers related to needs that facilitate the process of connecting to urban nature (see Supplementary Figure S1): (1) In the cluster “establishment/improvement of perceptibility,” 24 answers were identified. Female university students mentioned in this cluster that they need *breaks* to connect with urban nature, *no hustle and bustle and disturbances* as well as *no music but rather to listen to nature/animals/water*. (2) The cluster “no disturbing animals/other persons” included 10 answers. In this cluster, female university students stated that there should be *no tourists* and *insects* as they disrupt their ability to connect with urban nature and that *loneliness* is a vessel for urban nature connectedness. (3) The cluster “certain weather conditions, natural phenomena, seasons, and time of day” included four answers. Certain *seasons* (i.e., *spring and summer*), *convenient temperature* because it *determines length of exposure to nature* (*too hot versus too cold*), and *natural phenomena* (e.g., *sunset/sunrise, thunderstorm in summer, rainbows, snow*) were reported by female university students as being conducive to creating circumstances that better enable them to connect with urban nature. (4) In the cluster “leisure-time activities,” four answers were identified. Female university students mentioned in this cluster that a *vacation by the sea* helps promote urban nature connectedness. Furthermore, engaging in *activities in nature* (i.e., *painting, reading, making a campfire, camping, going on excursions with a stand-up paddle, kneipping, studying, and reflecting*) and *traveling to the not as densely populated countryside for a weekend* (e.g., *to a vacation home*) help cultivate urban nature connectedness. It was also suggested that *living in the not as densely populated countryside* facilitates urban nature connectedness, *as nature in the city is not comparable to nature in the countryside, particularly as a country person*. (5) The cluster “attractive design of the (inner) city” included 30 answers. In this cluster, female university students stated that they need *sand by lakes* (i.e., the *feeling of being by the sea*) and *natural shade from trees in summer* to establish urban nature connectedness and that *gray should be converted into green and not the other way around*. (6) The cluster “natural/regional food” included two answers. Female university students reported that *unprocessed food, especially at times when not being in nature much*, and *experiencing nature* by *buying food from local providers, farmers, or an organic farmer’s market instead of supermarkets* have a connective influence. As these avenues *mostly do not use plastic wrapping and factory farming*, they were perceived to enhance urban nature connectedness. (7) In the cluster “attractions,” 10 answers were identified. Female university students mentioned in this cluster that a *zoo* is beneficial for connecting with urban nature. Furthermore, *outdoor events* (e.g., *cinema in the park, gardening clubs*) and *mountains* (e.g., *to watch the sunrise/sunset, cows, etc*.) were also proposed as ways to enhance urban nature connectedness. (8) The cluster “accessibility” included seven answers. In this cluster, female university students emphasized the need to *travel through natural finish landscapes by public transport* to promote urban nature connectedness, *mobility without a car* (i.e., *cycling, walking, public transport*), and *animation for excursions* through *(connected) cycle paths to reach nature nearby and in the distance* with the *requirements of being simple, signposted, good, safe, and fast* [sic]. (9) The cluster “attractive design of the living space/environment” included seven answers. A *green (roof) terrace* (*with animals*), *indoor/useful plants* (e.g., *on window sill*), and an *apartment with a planted balcony and insects/birds to sit outside [sic]* were reported by female university students as ways to connect with urban nature. In addition, female university students mentioned that they *do not necessarily have to go outside* to connect to urban nature if they can *stay at home and enjoy a beautiful view through the window* (e.g., *mountains, sea, courtyards with plants/animals*).

#### 3.2.2 Ratings by female university students and dimensions of clusters

By 135 female university students, eight clusters were rated as unimportant on the four-point Likert scale, while only the cluster “accessibility” was rated as important (see Table 1). Overall, the clusters reflected the affective, experiential, cognitive, and behavioral attitude dimensions, with the spiritual dimension being the only one not represented.

**Table 1:** Importance ratings and dimensions of each cluster within the concept map of female university students (*N* = 135).

Dimension	Name of cluster	Importance rating (M ± SD)
Cognitive	Establishment/improvement of perceptibility	2.8 ± 0.9
Experiential	Attractive design of the (inner) city	2.7 ± 0.9
Experiential	Accessibility	3.0 ± 0.9
Experiential	Attractive design of the living space/environment	2.7 ± 0.9
Experiential	Leisure-time activities	2.9 ± 0.9
Experiential	Attractions	2.3 ± 0.9
Behavioral attitude, cognitive, and experiential	Natural/regional food	2.6 ± 1.0
Affective and cognitive	No disturbing animals/other persons	2.5 ± 0.9
Affective and experiential	Certain weather conditions, natural phenomena, seasons, and time of day	2.7 ± 0.9

M, Mean value; SD, Standard deviation.

## 4 Discussion

The aim of this concept mapping study was to identify what female university students from Southern Germany need to connect with urban nature. Almost all participants had access to nature, although slightly more than half lived in a city and only a minority lived in a rural area. They spent 8.6% of their weekly time in nature, distributed across more than half of the weekdays. In addition, the majority engaged in outdoor activities. Nevertheless, female university students reported a relatively low level of connectedness to urban nature. In total, nine different needs to connect to urban nature were identified among female university students. However, only “accessibility” was rated as important by them. Apart from the spiritual dimension, the clusters identified by the female university students reflected all dimensions of nature connectedness described in the introduction (i.e., affective, experiential, cognitive, and behavioral attitude).

### 4.1 Domains

In the following discussion, we have deductively categorized the identified needs into the three domains outlined in the review mentioned in the introduction, which grouped antecedents of nature connectedness into situational contexts, individual differences, and psychological states (30).

#### 4.1.1 Situational contexts

Situational contexts encompass experiences with nature that arise from actual contact, including where and when nature connectedness is promoted or inhibited (30). A total of seven of our identified clusters can be assigned to this domain, representing the presence of certain requirements that need to be fulfilled to foster connectedness in urban nature.

The fundamental need for “accessibility” is undeniable for having any chance of connecting with urban nature, which may explain why it was identified and rated as the only important need by female university students in the present study. This fundamental need corresponds to Target 11.7 of the United Nations’ Sustainable Development Goals (5). However, this finding should be interpreted with caution, as COVID-19 restrictions in 2021 may have influenced the results of this study. During COVID-19 in Southern Germany, movement and social restrictions were implemented (e.g., no day/group trips allowed), physical distancing regulations were enforced, facilities were closed (e.g., swimming pools, sports fields, zoos, campgrounds, public gardens and lakes, beer gardens), and events were prohibited (e.g., open-air cinemas, mountain and nature tours). As a result, access to urban nature was limited and, in some cases, impossible during this challenging time. Therefore, participants may have placed this need at the forefront of their thinking. In 2025, a study from the UK concluded that merely providing access to urban nature is not sufficient to support nature connectedness (46).

A previous study conducted in a residential rehabilitation center supports our finding that an “attractive design of the living space/environment,” such as views of nature or animals through windows and the presence of indoor plants, helps to foster nature connectedness (47). Indoor nature has previously been identified as a health-promoting method for urban inhabitants to connect with nature (48). The current study offers additional support for this feasible and affordable approach in daily life.

The relevance of an “attractive design of the (inner) city” for fostering nature connectedness, known as biophilic design, is also being researched (49). However, there is a need for design principles and robust evaluation (50) for this pathway to urban nature connectedness.

Previous research provides indications supporting our finding that (urban) nature connectedness is associated with engagement in “leisure-time activities” that take place in natural environments (51), such as outdoor activities (32). Interestingly, female university students also referred to behaviors outside the urban setting in this cluster. Traveling to the not-as-densely-populated countryside for a weekend (e.g., to a vacation home) might help urban inhabitants reconnect with nature; therefore, the limited nature available in the urban setting could still be sufficient to maintain this connectedness. However, living in the less densely populated countryside, as mentioned in this study, was found to be a negative predictor of urban nature connectedness in our previous research (32). This may be because nature in the city is not comparable to nature in the countryside, particularly for a country person.

Regarding the connection between “attractions”—a finding in our study—and nature connectedness, a previous study reported inconclusive evidence for zoos, as the results varied depending on whether an implicit or explicit measuring instrument was used (52). However, mountains have been identified as a positive feature (46). In clear weather, the Alps can be seen from hills and roof terraces in the city where the study was conducted (e.g., for watching the sunrise/sunset). This might explain why female university students mentioned this city-specific feature.

Our findings in the cluster “certain weather conditions, natural phenomena, seasons, and time of day” are in line with a previous study showing an association between seasonal and meteorological factors and implicit nature connectedness (53). This may suggest that female university students favor conditions that do not impact their feminine beauty ideals when going outside (e.g., impact of sweating on outward appearance when it is too hot). A previous study from the United States supports this suggestion by confirming a theoretical model in which self-objectification and internalization of the feminine beauty ideal negatively impact nature connectedness in female individuals by limiting their experiences in nature (54). This limitation of experiences in nature was also observed in another study from the United States, which found that more female individuals than male individuals preferred to exercise indoors in winter (55), possibly due to cold temperatures. As female individuals generally spend less time outdoors compared to male individuals, there seems to be an inequality in this regard (56). This gender gap in being outdoors appears relevant to Goal 5 of the United Nations’ Sustainable Development Goals (5), as the benefits of nature may be less accessible to female individuals. Our findings—that female university students are attracted to natural beauty, including natural phenomena such as sunset/sunrise or rainbows, favor spring as a preferred season, and prefer convenient temperatures (i.e., mild summer or not too cold conditions)—further support this observation.

We contribute to closing a research gap identified in a previous review on the antecedents of nature connectedness (30) by elaborating on a set of conditions during contact with nature that actively inhibit nature connectedness, as highlighted in the cluster “no disturbing animals/other persons”. This cluster includes comparison conditions by contrasting living beings that promote urban nature connectedness (such as sympathetic and considerate dog owners that clean up their dog’s excrement and put a leash on their dog) with living beings that inhibit urban nature connectedness (such as tourists and insects). The reason why female university students identified this cluster may be that they tend to prefer using outdoor environments for recreation (51). Therefore, they do not want to be distracted, as such distractions could reduce the quality and enjoyment of their nature experience. This finding is particularly noteworthy, as most people usually report positive factors associated with nature connectedness (57).

#### 4.1.2 Individual differences

The individual differences domain refers to relatively stable characteristics, such as demographics (e.g., age, sex, race, socioeconomic status), personality traits, and worldviews, including beliefs, attitudes, orientations, and values (30).

As it reflects worldviews, we assign the cluster “natural/regional food” to this domain. This finding is in line with previous research showing implicit nature connectedness through food (58). It is not surprising that female university students identified this cluster. This cluster aligns with health-related behaviors more common among female individuals, such as buying organic food more often than male individuals (59), as well as with environmental attitudes, such as using less plastic packaging (60) and supporting animal welfare (61).

#### 4.1.3 Psychological states

The domain of psychological states includes psychological processes related to mindfulness, the self, affect, and motivation (30).

To this domain, we assign the cluster “establishment/improvement of perceptibility,” as identified in female university students, because it includes strategies that promote mindfulness (e.g., taking breaks, avoiding music and instead listening to nature, animals, or water, and avoiding hustle, bustle, and disturbances). According to a previous meta-analysis, mindfulness is related to nature connectedness (62).

### 4.2 Strengths and limitations

The main strength of this mixed methods study is its large sample size of 152 female university students, which far exceeds the typical recommendation of 15 participants for concept mapping (63) and enhances the relevance of our findings, as we can assume that data saturation was achieved. In contrast, the main limitations of this study relate to the sample: (a) Due to varying contexts, the generalizability of our results from Germany to other countries may be restricted. (b) There is a selection bias from using a convenience sample of university students, influenced by the COVID-19 pandemic. (c) Male university students were excluded from the analysis because of their small number and the imbalanced distribution between the sexes. For this reason, potential similarities and differences between sexes in terms of their needs to connect with urban nature remain unknown. (d) As the socioeconomic status of participants was not assessed due to inconclusive evidence of its effect on nature connectedness (30), no conclusion could be drawn regarding this factor in our study. The nature of our methodological approach introduces the possibility of response bias, as participants may have felt obliged to provide answers. According to the Mixed Methods Article Reporting Standards (64), another limiting factor is that mixed methods research includes a qualitative component, which is at high risk of reflexive bias. However, efforts were made to minimize this bias by addressing the interviewer effect. Therefore, data were collected and analyzed by a researcher or assistant of the same sex as the participants (i.e., a female researcher or assistant supervised the female university students). In addition, we sought to ensure robustness by analyzing the data in consultation with at least one other reviewer. It was deemed unfeasible to conduct participant-led group discussions to reach consensus on data interpretation due to the large sample size and the challenges at the time of the study, which necessitated an online format for data collection.

## 5 Conclusion

By providing meaningful insights into the needs of a considerable sample of female university students regarding nature connectedness in the urban setting, this mixed methods study from Southern Germany is unique and makes a significant contribution to the research field. Our study helps understand how urban nature connectedness is developed and how this construct could be defined on the basis of content validity, as supported by our findings. We show that four of the five dimensions (i.e., affective, experiential, cognitive, and behavioral attitude) and all three domains of nature connectedness (i.e., situational contexts, individual differences, and psychological states) are also relevant for urban nature connectedness among female university students. This suggests that the constructs of (urban) nature connectedness might not be that different from each other. Given that the experiential dimension and the situational contexts domain predominate in our study, urban nature connectedness may be considered to be a state rather than a trait. Furthermore, our findings indicate that a mere summary of needs—without considering the importance of factors—is not sufficient to draw solid conclusions for practical implications. Finally, by identifying a need in female university students that highlights factors inhibiting nature connectedness in the urban setting, we might inspire future research on the barriers to (urban) nature connectedness.

We recommend that future research on the operationalization of the multidimensional and complex construct of urban nature connectedness, the development and validation of a robust measuring instrument, and the development of an intervention to promote urban nature connectedness should consider our findings directly obtained from the target group. By involving the target group as experts from the beginning and incorporating their various perspectives based on their own understanding of the construct, the gap between theory and practice could be bridged. In addition, our findings can guide policymakers and city planners on how to optimize urban design to improve urban nature connectedness for the promotion of health among urban inhabitants. The findings of our study could be complemented by perspectives from male university students for a sex/gender analysis, as well as input from other experts, such as practitioners connected to nature (65) (e.g., writers, photographers, filmmakers, activists, and gardeners) and researchers, to counteract unconscious bias. In future research, our methodological approach could also be applied to different age groups (i.e., children, adolescents, and older adults) and—in line with our previous findings (32)—to different residential areas and populations spending differing amounts of time outdoors in nature for multiple purposes. Finally, future research could investigate how fast and to what extent urban nature connectedness is developed if needs are satisfied, as well as the long-term impact of need satisfaction on the maintenance of urban nature connectedness.

## Data Availability

The raw data supporting the conclusions of this article will be made available by the authors, without undue reservation.
