# Supplementary material for: Needs to connect to urban nature in female university students from Southern Germany: a mixed methods concept mapping study
Source: Front Public Health. 2026 Mar 17;14:1758383. doi: 10.3389/fpubh.2026.1758383 (PMC13036136; doi:10.3389/fpubh.2026.1758383)
Supplement: Supplementary file 1 [file Table_1.DOCX]

**S1 Table. Personal and warm-up questions answered by female university students during the first concept mapping session (*N* = 152).**

| **Survey item** |  |
| --- | --- |
| Age (in years) | 22.7 ± 2.8 |
| Residential area  a) rural  b) small town  c) medium-sized town  d) city | 17 (11.3%)  23 (15.3%)  26 (17.3%)  84 (56.0%) |
| Marital status  a) single  b) in a relationship  c) married | 71 (46.7%)  77 (50.7%)  4 (2.6%) |
| Parental Status  a) yes = have children  b) no = do not have children | 5 (3.3%)  147 (96.7%) |
| Extent of media use (in hours per day) | 6.0 ± 2.6 |
| Religious affiliation  a) yes  b) no | 76 (50.0%)  76 (50.0%) |
| Dog ownership  a) yes  b) no | 16 (10.5%)  136 (89.5%) |
| Access to nature  a) yes  b) no | 150 (98.7%)  2 (1.3%) |
| Time spent in nature (in days per week) | 4.5 ± 1.8 |
| Time spent in nature (in hours per week) | 14.4 ± 10.6 |
| Engagement in outdoor activities  a) yes  b) no | 133 (87.5%)  19 (12.5%) |
| Urban nature connectedness  (1 = not at all, 2 = rather not, 3 = rather yes, 4 = very much) | 2.8 ± 0.7 |
