# Supplementary material for: Needs to connect to urban nature in female university students from Southern Germany: a mixed methods concept mapping study
Source: Front Public Health. 2026 Mar 17;14:1758383. doi: 10.3389/fpubh.2026.1758383 (PMC13036136; doi:10.3389/fpubh.2026.1758383)
Supplement: Supplementary file 2 [file Data_Sheet_1.DOCX]

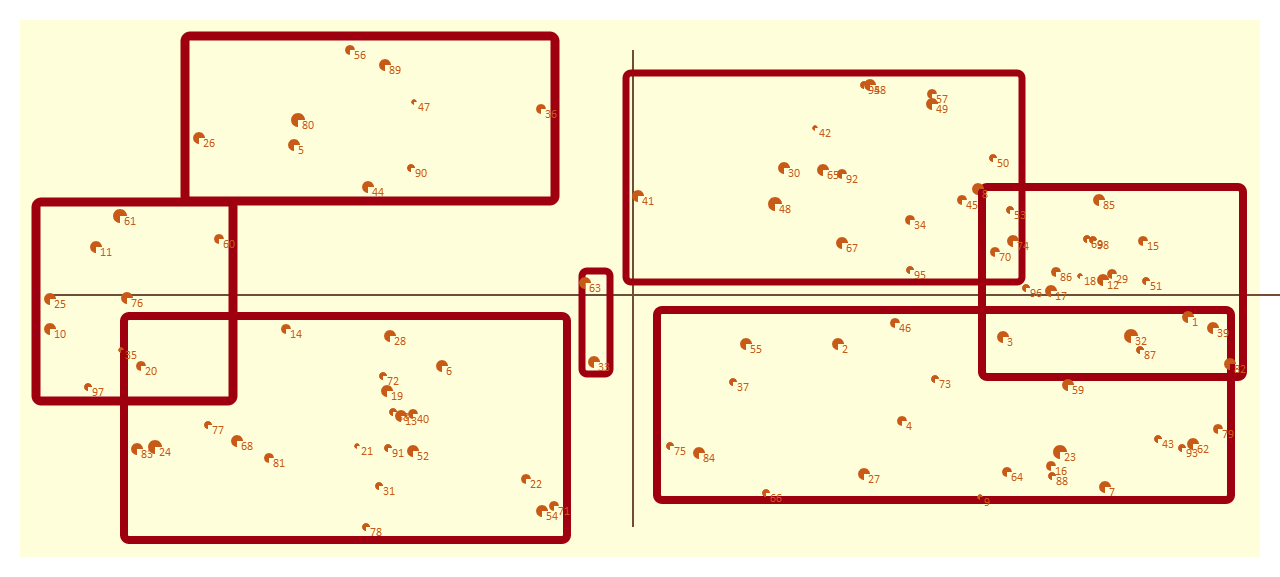


6

8

7

1

4

9

5

2

3

**S1 Figure.** **Concept map of female university students (*N* = 139).** Clusters: (1) establishment/improvement of perceptibility, (2) no disturbing animals/other persons, (3) certain weather conditions, natural phenomena, seasons, and time of day, (4) leisure-time activities, (5) attractive design of the (inner) city, (6) natural/regional food, (7) attractions, (8) accessibility, and (9) attractive design of the living space/environment. An original cluster is indicated by a square and a newly created cluster by a circle. A reallocation of an answer (arranged as a dot) into another cluster is indicated by an arrow. The axes represent the vertical dimension top/bottom and horizontal dimension left/right as the imaginary distance between answers represents their correlation in a two-dimensional coordinate system.
